# The biomechanical role of meniscal allograft transplantation and preliminary in-vivo kinematic evaluation

**DOI:** 10.1186/s40634-019-0196-2

**Published:** 2019-06-25

**Authors:** Stefano Zaffagnini, Stefano Di Paolo, Federico Stefanelli, Giacomo Dal Fabbro, Luca Macchiarola, Gian Andrea Lucidi, Alberto Grassi

**Affiliations:** 1 IIa Clinica Ortopedica e Traumatologica, IRCCS Istituto Ortopedico Rizzoli, Bologna, Italy; 2 Dipartimento di Scienze Biomediche e Neuromotorie DIBINEM, Università di Bologna, Bologna, Italy

**Keywords:** Meniscus, Knee, Meniscus allograft transplantation, Biomechanical evaluation, Laxity test

## Abstract

**Background:**

Meniscus allograft transplantation (MAT) is a surgical procedure performed in patients complaining post-meniscectomy syndrome. Although the effectiveness of MAT on knee stability has been already demonstrated in cadaveric studies, its biomechanical role has been poorly evaluated in-vivo.

**Methods:**

A narrative review of the biomechanical effect of meniscectomy and MAT was performed. Furthermore, two cases were presented, of one patient who underwent Medial MAT and Anterior Cruciate Ligament (ACL) reconstruction, and one who underwent Lateral MAT. During the surgery, knee laxity was evaluated using a surgical navigation system.

**Results:**

AP laxity and IE rotation were reduced of 25% to 50% at both 30° and 90° of knee flexion after MAT transplantation.

**Discussion:**

In both cases, almost all the tests performed showed a reduction of knee laxity after meniscus transplant, when compared with pre-operative knee laxity. This assessment confirms the insights of previous in-vitro studies and underline a crucial role of MAT in knee biomechanics.

## Background

Menisci are an important structure of the knee and strongly contribute to different functions, such as load distribution, secondary stabilization of the knee, and tibiofemoral congruity (Ahmed & Burke, 1983; Haut Donahue et al., 2004; Levy et al., 1989; Levy et al., 1982; Markolf et al., 1981) . Meniscal lesions are one of the commonest injury worldwide, with an annual incidence of 60–70 per 100,000 inhabitants (Beals et al., 2016). Despite the common increasing trend of preserving the meniscus through sutures and repair, the meniscectomy is still the treatment of choice (Jacquet et al., 2019). Consequences of meniscectomy have been widely evaluated in the past.

Different studies demonstrated a correlation between meniscectomy and knee degeneration (Allen et al., 1984; Faunø & Nielsen, 1992; Scheller et al., 2001).

In long term follow-up (up to 22 years) 27% of patients who underwent meniscectomy developed symptomatic radiographic knee OA (corresponding to Kellgren/Lawrence grade > or = 2), with a relative risk of 2.6 times higher than the contralateral non-operated knee (Englund & Lohmander, 2004).

A systematic review performed by Petty (Petty & Lubowitz, 2011) found an higher rate of joint degeneration, up to 53%, compared with contralateral uninjured knee.

Meniscectomy is also associated with worse clinical outcomes, such as Lysholm scores, Tegner Activity Level, instability, and removal of lateral meniscus lead to increased instability and poor outcomes (Salata et al., 2010).

Meniscus Allograft Transplantation (MAT) has been proposed as a surgical option to treat patients with symptomatic total or subtotal meniscectomies, with the aim of reducing pain and improving knee function. Its satisfactory clinical results has been demonstrated in more than 40 studies and 1500 patients, (Rosso et al., 2015) often in combination with Anterior Cruciate Ligament (ACL) reconstruction. (Saltzman et al., 2017) Clinical scores confirm the effectiveness of MAT in contrasting the progression of knee osteoarthritis (Young et al., 2017). In patients with significantly arthritic knees, MAT in conjunction with articular cartilage repair may help to delay further surgical treatment by an average of 5 years (Stone et al., 2010).

However, MAT has also been suggested to have a relevant role in controlling knee laxities and thus protecting the ACL (Novaretti & Musahl, 2018). Nevertheless, the biomechanical effect of MAT has been mainly evaluated through cadaveric studies, and there is lack of in-vivo evaluations.

In this study, we first aim to review the biomechanical consequences of meniscectomy and MAT on knee stability. In order to do so, we focused on two main biomechanical parameters: contact stress, since its increase can lead to early OA (Dong et al., 2014); and knee laxity, since it is one of the principal indices of knee instability. Furthermore, we aim to offer an insight about the kinematic effect of either medial or lateral MAT, through an in-vivo kinematic acquisition with a navigation system.

### Biomechanical consequence of meniscectomy

#### Contact stress

The weight-bearing role of the meniscus has been investigated in past (Maher et al., 2017). A greater attention has been focused on the effect of the either partial or total removal of the medial meniscus, since it is the mostly solicited during daily life activities.

Overall, the internal forces acting on the knee after meniscectomy significantly differ from the intact conditions. Knee biomechanics may result in an alteration of contact pressure and contact area. Baratz et al. (Baratz et al., 1986) showed a proportional increase of contact pressure (up to 110%) after partial-to-total meniscectomy in cadaver knees. In addition, contact area has been shown to decrease significantly (up to 75%) relating to the size of the damage. In particular, the peripheral portion of the menisci seems to contribute most to the changes in the knee contact stress (Lee et al., 2006).

As abnormal loading conditions may affect the integrity of the cartilage, meniscectomy is commonly considered as a risk factor for the beginning and the progression of osteoarthritis (Petty & Lubowitz, 2011). The direct contact between the cartilage layers may facilitate the damage of the collagen matrix and prolong the strain recovery. Thus, this may induce vascular invasion, dehydration and endochondral ossification (Song et al., 2008).

#### Laxity

The menisci have an important role in static and dynamic knee laxity. Numerous in vitro studies highlighted the importance of the menisci in reducing anterior tibial translation in the knee in different ACL conditions. (Allen et al., 2000; Hanley & Warren, 1987; Levy et al., 1989; Levy et al., 1982)

Removal of the medial meniscus has been reported to produce an increase of the strain on the ACL and to contribute to anterior-posterior (AP) laxity, when the ACL is intact (Spang et al., 2010). Compared to the normal condition, removal of almost 50% of the posterior horn of the medial meniscus increases anterior tibial translation and creates a posterior shift of the femur under axial compression (Arno et al., 2013). Through an in-vivo study, Yammine et al. (Yammine, 2013) showed how partial meniscectomy may induce significant immediate post-operative anterior tibial translation (up to 3 mm) even when ACL is not injured.

In an ACL-deficient knee, the effect of medial meniscus injury has been widely studied. A commonly accepted insight is that posterior horn tears (Ahn et al., 2011) or posterior root tears increase AP tibial translation, especially when the knee is flexed 0–60 degrees.

Lorbach et al. (Lorbach et al., 2015) demonstrated that partial or total medial meniscectomy significantly altered AP translation and pivot-shift in the ACL-deficient knee in cadaveric specimens, while meniscal repair effectively restored the intact meniscus status.

A recent study by DePhillipo et al. (DePhillipo et al., 2018) highlighted that ramp lesions increase anterior tibial translation, IE rotation, and Pivot-shift in an ACL-deficient knee. After isolate ACL reconstruction, Pivot shift was not completely restored.

The effect of lateral meniscectomy on AP laxity has been inspected less, and mainly in cadaveric studies: Wieser et al. (Wieser et al., 2012) did not find any statistical difference after meniscus removal in stable knees; using computer navigation, Musahl et al. (Musahl et al., 2010) confirmed the limited effect of the lateral meniscus in resisting anterior tibial translation in the ACL-deficient knee. However, the same authors demonstrated that total lateral meniscectomy in an ACL-deficient knee increased anterior translation of the lateral compartment during the pivot-shift maneuver. A similar effect has also been noted after lateral meniscus posterior root tear (Frank et al., 2017; Shybut et al., 2015) .

The combined effect of ACL replacement and meniscectomy has also been evaluated. Seon et al. (Seon et al., 2009) reported a residual AP laxity (7 mm) after single-bundle ACL reconstruction if subtotal medial meniscectomy was performed, compared with intact menisci. In particular, this was mostly evident at high degrees of knee flexion. In an in vitro study, Papageorgiou et al. (Papageorgiou et al., 2001) reported increased in situ forces in the ACL graft between 30% and 50% after medial meniscectomy in response to a combined anterior ad axial load, which could theoretically increase the risk of graft failure. Moreover, during a mechanized pivot shift, increased anterior translation of the lateral compartment with respect to the intact knee was demonstrated when both menisci were removed during ACL reconstruction (Petrigliano et al., 2011).

### Biomechanical effect of MAT

#### Contact stress

MAT has been reported to be effective in the treatment of meniscus injury, and to partially restore the biomechanical function of the knee after the meniscectomy (Seitz & Dürselen, 2018). An in-vitro study by Kim et al. (Kim et al., 2013) demonstrated that joint contact pressure in meniscectomized knees were significantly higher than pressure after MAT, especially at 30° and 60° of knee flexion. Similarly, McDermott et al. (McDermott et al., 2008) showed that joint contact pressure after MAT are close to the ones in the intact knee, after being significantly risen in knees with medial meniscectomy. These results confirmed the potential chondroprotective effect of MAT in knee osteoarthritis.

#### Laxity

To the date, the biomechanical effect of MAT on knee laxity has been poorly evaluated (Rosso et al., 2015). An interesting in-vitro assessment of knee stability in presence of meniscal and ACL lesion has been given by Musahl et al. (Musahl et al., 2010): Lachman and Pivot-shift test have been used to evaluate the AP laxity when either medial or lateral meniscus were removed after the simulation of an ACL-deficient condition. A subsequent study from the same authors (Novaretti & Musahl, 2018) demonstrated that, in the same clinical conditions, lateral MAT may partially reduce AP laxity with both a suture-only and a bone-block technique (approximately 50% less than meniscectomized knee). Through another in-vitro study, Spang et al. (Spang et al., 2010) also assessed the effect of MAT in reducing the anterior tibial translation, demonstrating that laxity was statistically restored to the intact conditions. Nevertheless, the stability of the intact knee was not restored. In these studies, a surgical navigation system has been used to evaluate the intra-operative knee kinematics on cadavers. So far, no studies have outlined the in-vivo effect of MAT on knee kinematics.

### In-vivo biomechanical evaluation of MAT

#### Patient 1 – medial MAT

##### Patient presentation

When the patient came to authors attention, he was a 55 years old male complaining of severe medial compartment pain of the right knee during working, walking and playing sports for 2 years and sensation of knee instability during pivoting movement. The patient was a heavy worker, amateur sportsman, with a Body Mass Index (BMI) of 24 kg/m^2^, healthy and without relevant comorbidities. The patient had undergone arthroscopic subtotal medial meniscectomy of the right knee 25 years before surgery, due to a traumatic lesion occurred during sport activity. He was asymptomatic until 5 years ago, when he started to complain worsening medial compartment tenderness, however without limiting his normal activities. Pain and swelling became more severe in the last 2 years to preclude him any sport activity such as running and playing tennis. The patient also reported a recent traumatic right knee sprain 6 months before the visit treated conservatively, after which a sense of knee instability and giving way, especially during pivoting activities, made him look for medical attention.

During the visit the patient presented a positive joint line tenderness at palpation, Anterior drawer test 3+ (scale from 0+ to 3+), Lachman test 3+ (scale from 0+ to 3+), Pivot-Shift test 2+ (scale from 0 to 3+), negative Varus and Valgus stress test (Mulligan et al., 2015; Musahl et al., 2012).

**Fig. 1:**
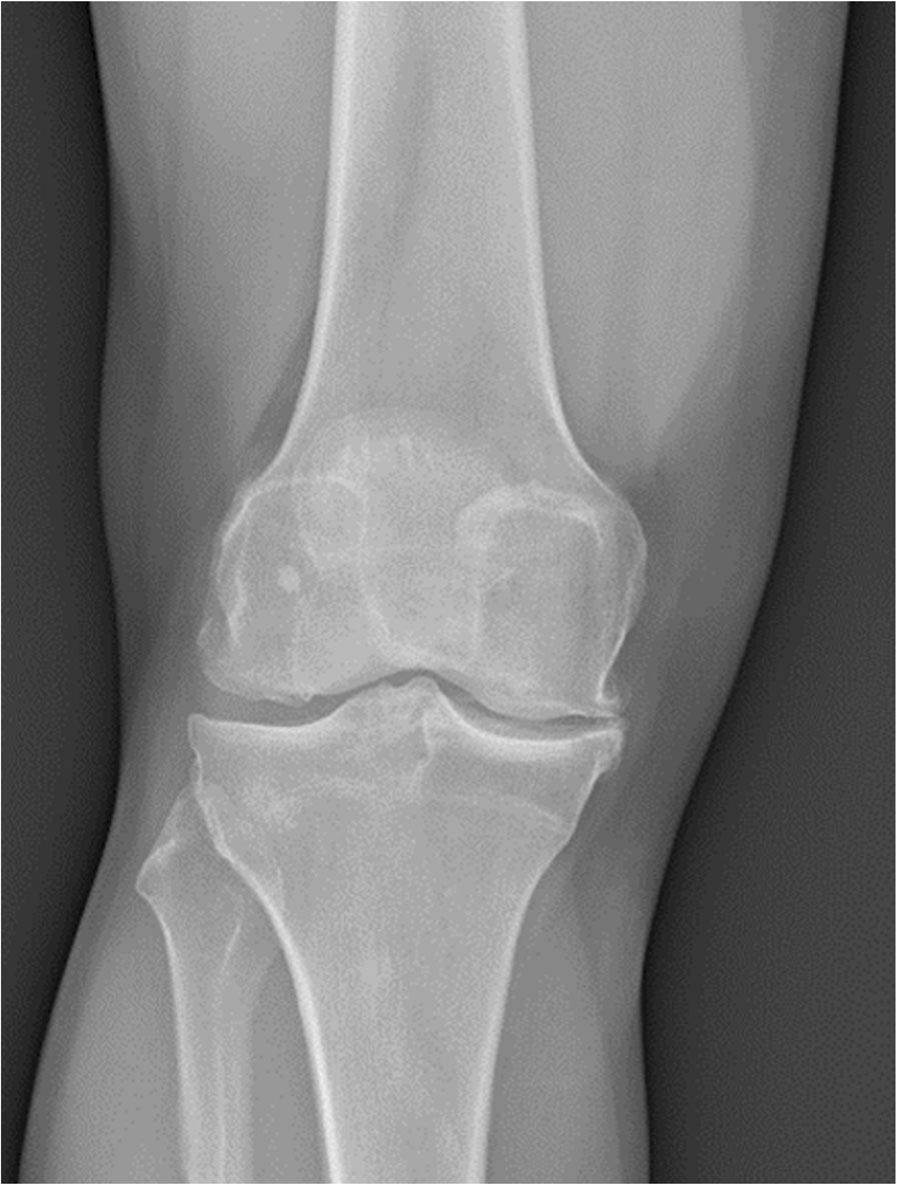
Pre-operative AP knee radiography Patient 1

Knee radiographs revealed joint space narrowing and small osteophytes of medial femoral condyle and medial tibial plateau (Kellgren-Lawrence grade 3) (Fig. 1) (Kohn et al., 2016).

**Fig. 2:**
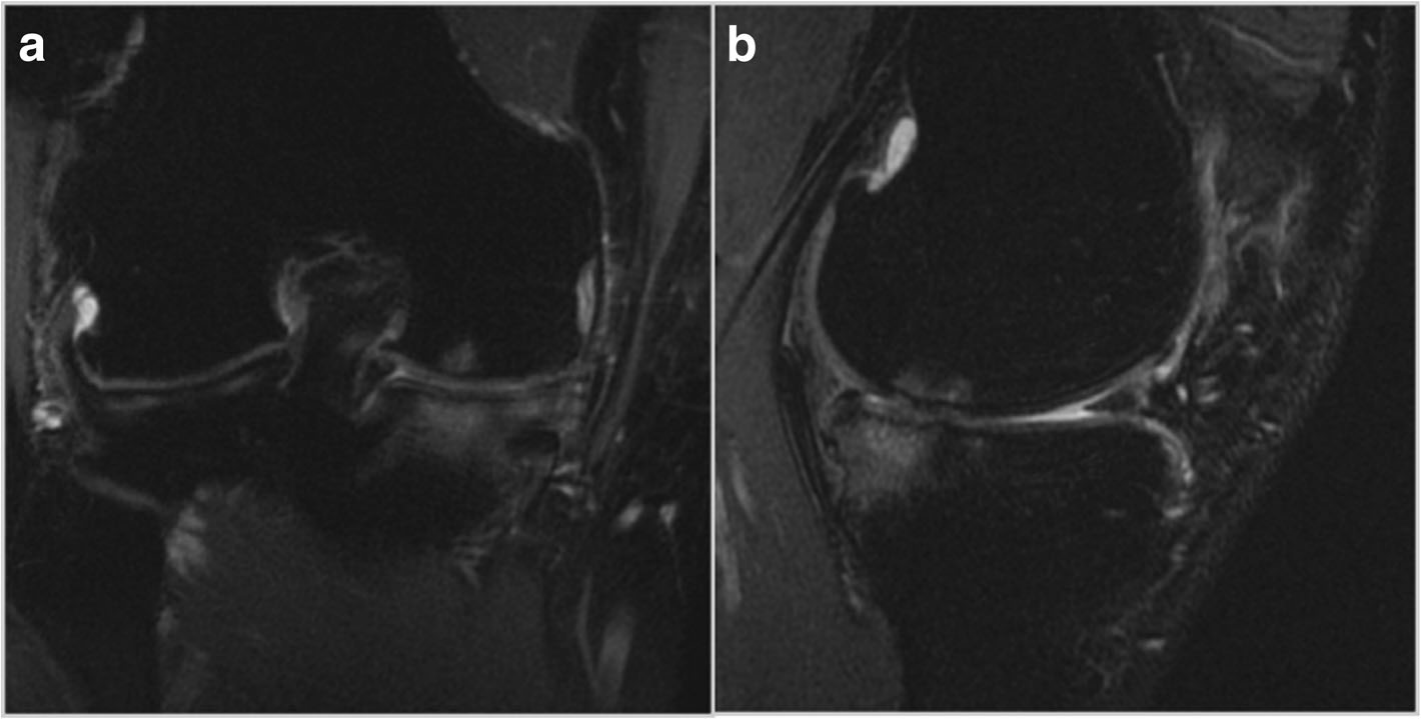
A recent frontal (**a**) and sagittal (**b**) MRI Patient 1

The Magnetic Resonance Imaging (MRI) showed absence of continuity and dishomeogenous signal of ACL, subtotal medial meniscectomy and subchondral bone edema of medial femoral condyle and tibial plateau, with a chondropathy graded as II according to Yulish Classification (Fig. 2) (Yulish et al., 1987).

Posterior Cruciate Ligament (PCL), lateral meniscus, lateral and patelloframroal compartments did not presented relevant abnormalities**.**

After counseling, the patient was scheduled for combined ACL reconstruction and medial MAT of the right knee.

##### Surgical procedure

**Fig. 3:**
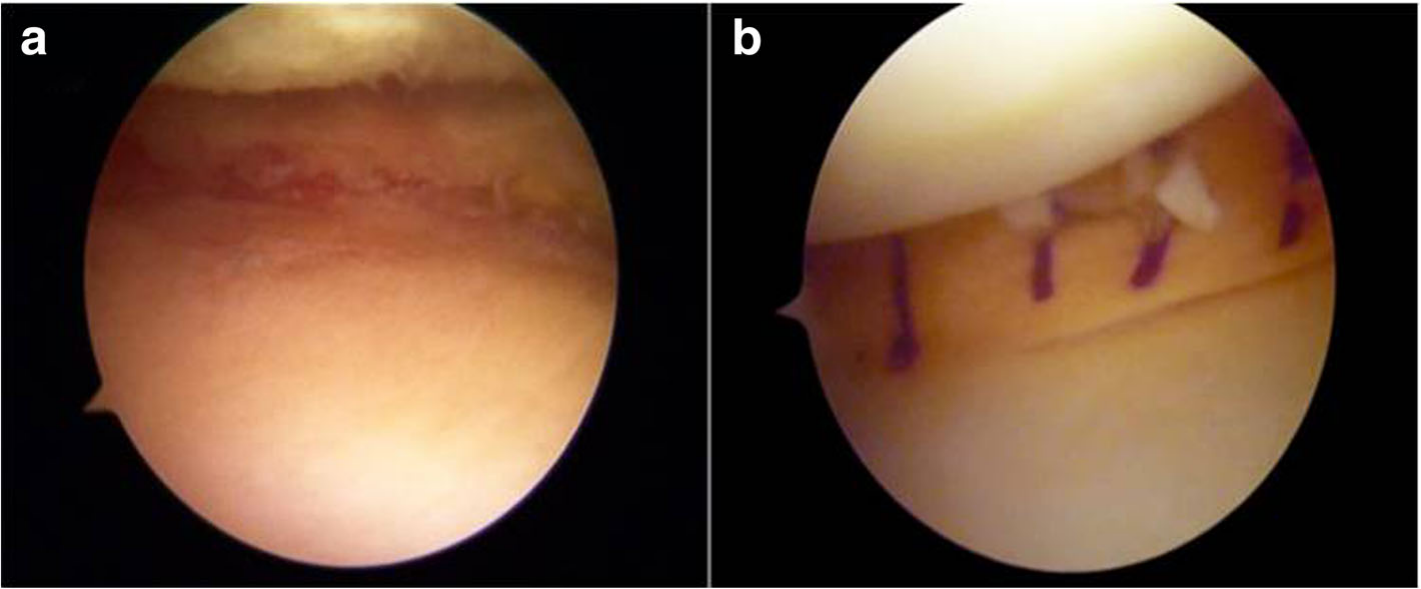
Arthroscopic evaluation: (**a**) Pre-operative and (**b**) after medial MAT Patient 1

Arthroscopically, chronic ACL lesion with degeneration of the stump was noted. Lateral compartment presented an Outerbridge grade I of the lateral femoral condyle, with no lateral meniscus lesions. The medial compartment presented a subtotal deficit of medial meniscus, an Outerbridge grade II of the medial femoral condyle and a grade III of the medial tibial plateau (Fig. 3a).

Before the MAT and ACL reconstruction, kinematic acquisition was performed using a navigation system (BLU-IGS, Orthokey, Lewes, Delaware, DE, USA) equipped with a dedicated software (KLEE, Orthokey, Lewes, Delaware, DE, USA). Two optical trackers were firmly fixed at femoral and tibial bone through stab incisions, and anatomical landmarks were registered with a third tracker. After the initial set-up, the surgeon manually performed a battery of clinical kinematic tests at maximum manual force (Table 1). The reliability of all the laxity tests performed was evaluated by the research group in previous studies (Lopomo et al., 2009; Martelli et al., 2007).After the tests, arthroscopic meniscal allograft transplantation was performed, according to Marcacci’s technique. (Marcacci et al., 2012) A non-irradiated medial meniscus allograft was prepared without bone plugs. The graft was inserted in the knee joint through arthroscopic portal and the posterior horn was fixed to the anterior tibia through a trans-osseous suture, the periphery was sutured to the capsule with all-inside TRUESPAN (Mitek Sports Medicine, Raynham, Massachusetts, MA, USA), stitches, while the anterior horn was fixed to the remnant of the native meniscus with an all-inside stitch and to the capsule with a free needle (Fig. 3b).

**Table 1:** Kinematic tests performed

Anterior/posterior displacement at 30° of flexion (AP30);	
Anterior/posterior displacement at 90° of flexion (AP90);	
Internal/external rotation at 30° (IE30);	
Internal/external rotation at 90° (IE90);	
Varus/valgus test at 0° (VVO);	
Varus/valgus test at 30° (VV30);	

After the transplant, knee laxity was again evaluated with the same tests through surgical navigation system.

Then, ACL reconstruction was performed with a single-bundle Over the top plus lateral plasty technique, using hamstring graft without detaching their tibial insertion (Marcacci et al., 1998).

Finally, the same laxity tests were performed after graft fixation. All the laxity tests were performed by the same experienced surgeon at manual maximum load.

##### Results

**Fig. 4:**
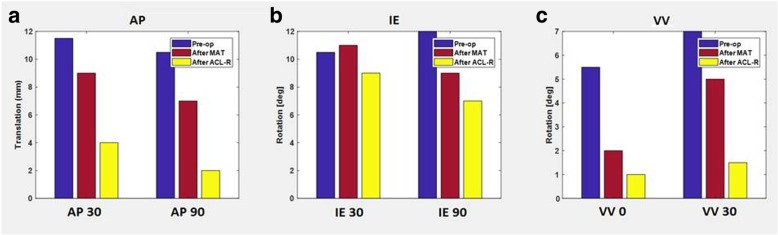
Laxity evaluation: AP30 and AP90 (**a**), IE30 and IE90 (**b**), VV0 and VV30 (**c**) for Medial MAT

AP displacement at 30° of flexion was 11.5 mm, 9 mm and 4 mm, at the basal state, after MAT and after ACL reconstruction, respectively. AP displacement at 90° of flexion was 10.5 mm, 7 mm and 2 mm, at the basal state, after MAT and after ACL reconstruction, respectively. Varus-Valgus (VV) rotation at 0° of flexion was 5.5°, 2° and 1°, at the basal state, after MAT and after ACL reconstruction, respectively. Internal-External (IE) rotation at 30° of flexion was 10.5°, 11° and 9°, at the basal state, after MAT and after ACL reconstruction, respectively, while the IE rotation at 90° of flexion was 12°, 9° and 7°, at the basal state, after MAT and after ACL reconstruction, respectively VV rotation at 30° of flexion was 7°, 5° and 1.5°, at the basal state, after MAT and after ACL reconstruction, respectively (Fig. 4).

## Patient 2 – lateral MAT

### Patient presentation

The patient was 30 Years old, amateur sportsman, with BMI 25,3 kg/m^2^, standing worker, without relevant comorbidities. 12 years before he underwent arthroscopic subtotal lateral meniscectomy of the left knee due to a traumatic meniscal lesion occurred while he was playing basketball. After surgery, he continued playing basketball and other pivoting sports, such as rugby and ski, for several years. In the last 4 years, he started complaining pain in the lateral compartment of the knee, he stopped playing pivoting sports and started bike, swim and running. In the last two years, he stopped playing sports because of pain increase and knee swelling episodes.

During the visit patient presented a tenderness in the lateral joint line of the knee. Laxity tests were negatives for ligamentous lesions.

**Fig. 5:**
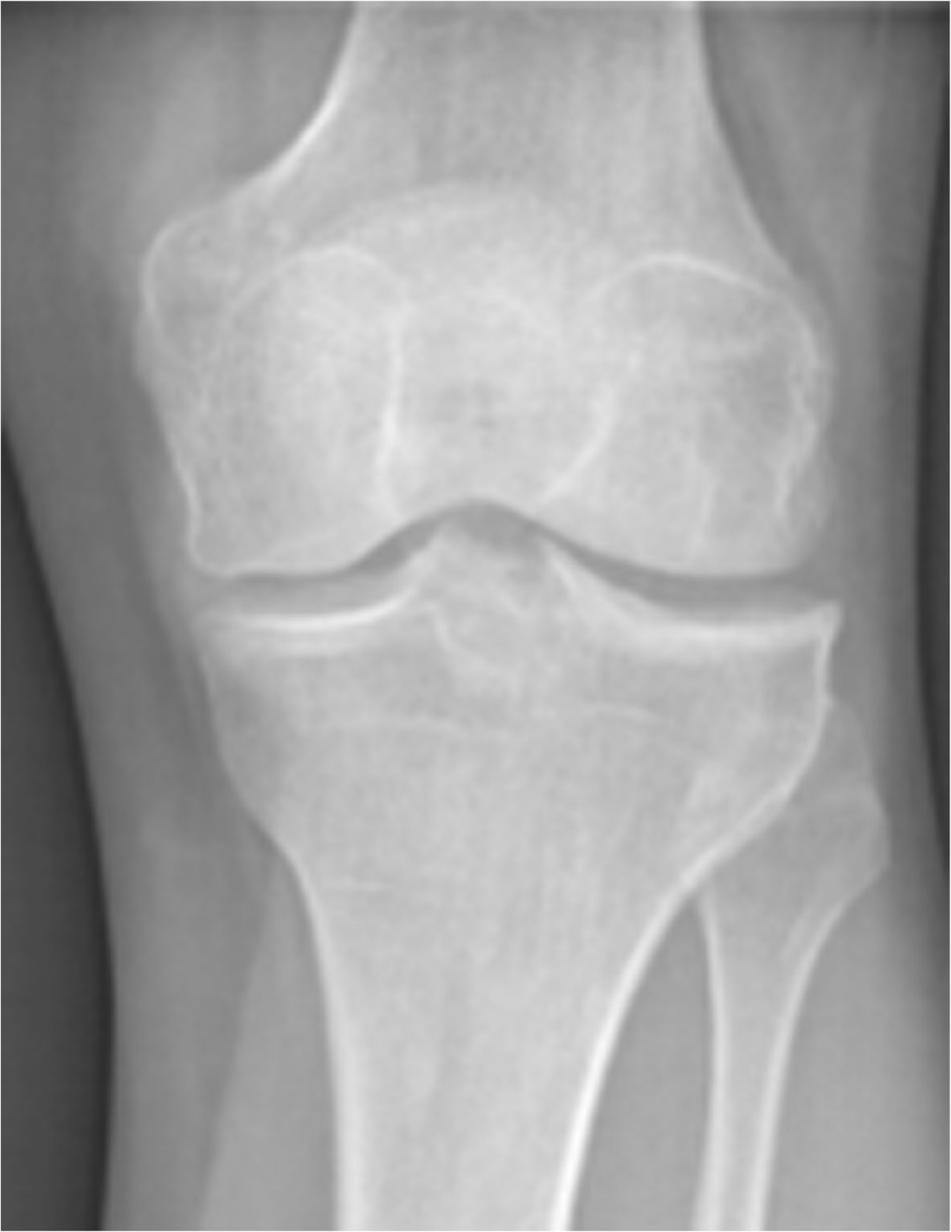
Pre-operative AP knee radiography Patient 2

Knee radiographs showed initial lateral joint space narrowing, graded as I according to Kellgren-Lawrence classification (Fig. 5).

**Fig. 6:**
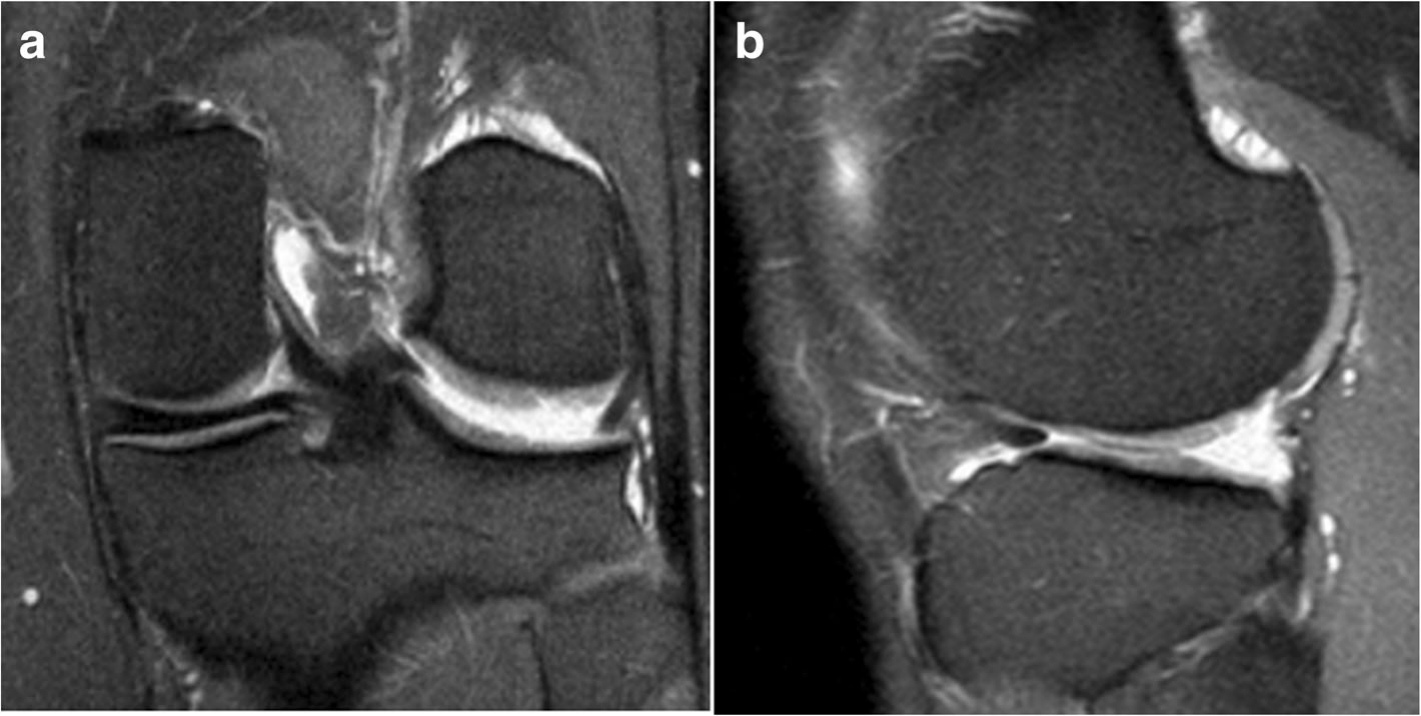
A recent frontal (**a**) and sagittal (**b**) MRI Patient 2

The MRI showed subtotal lateral meniscectomy and a chondropathy graded as II according to Yulish classification. Other structures did not present relevant abnormalities (Fig. 6).

The patient was scheduled for arthroscopic lateral MAT of the left knee.

### Surgical procedure

**Fig. 7:**
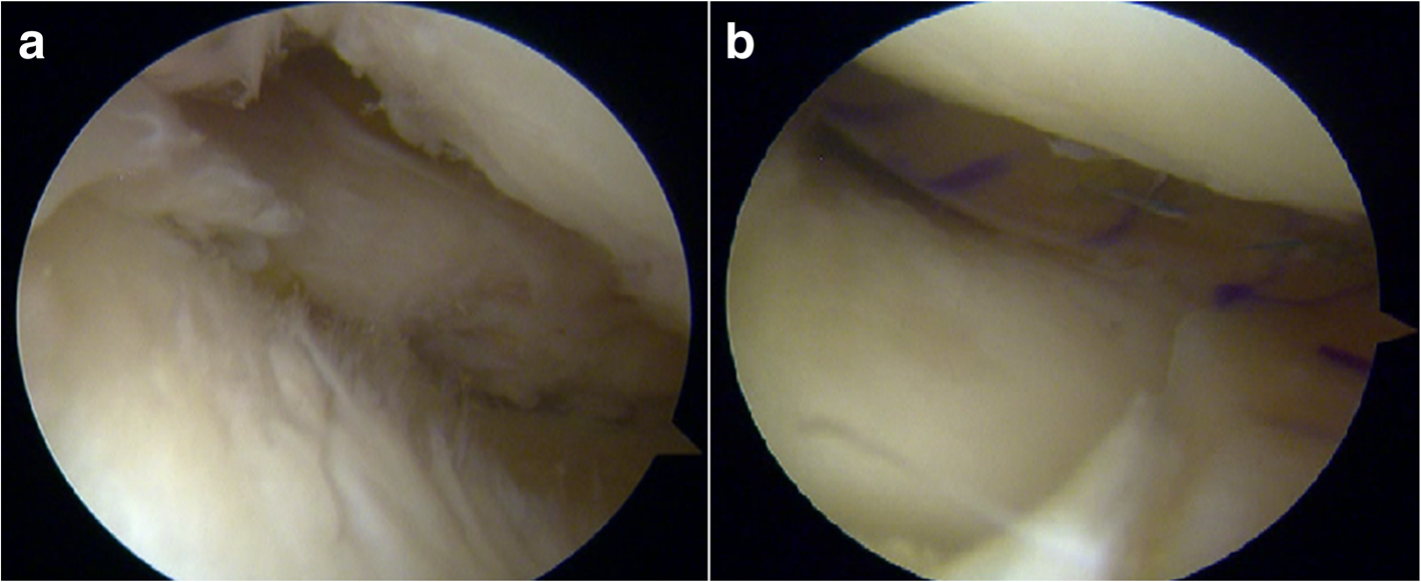
Arthroscopic evaluation: (**a**) Pre-operative and (**b**) after lateral MAT Patient 2

Anteromedial and anterolateral arthroscopic portal were performed. Arthroscopically, the medial compartment, ACL and PCL did not show significant lesions. The lateral compartment presented a subtotal deficit of lateral meniscus and an Outerbridge grade II of the lateral femoral condyle. (Fig. 7a).

Before MAT, kinematic tests were performed and evaluated with navigation system.

Then, arthroscopic lateral meniscal allograft transplantation was performed with a bone plug-free technique, fixing the anterior and posterior horns through two trans-tibial tunnels, while the periphery was sutured to the capsule with all-inside stitches FasT-Fix (Smith & Nephew, Andover, MA, USA) (Zaffagnini et al., 2016) (Fig. 7b).

After graft fixation, new laxity tests were performed and evaluated through surgical navigation system. All the laxity tests were performed by the same experienced surgeon at manual maximum load.

### Results

**Fig. 8:**
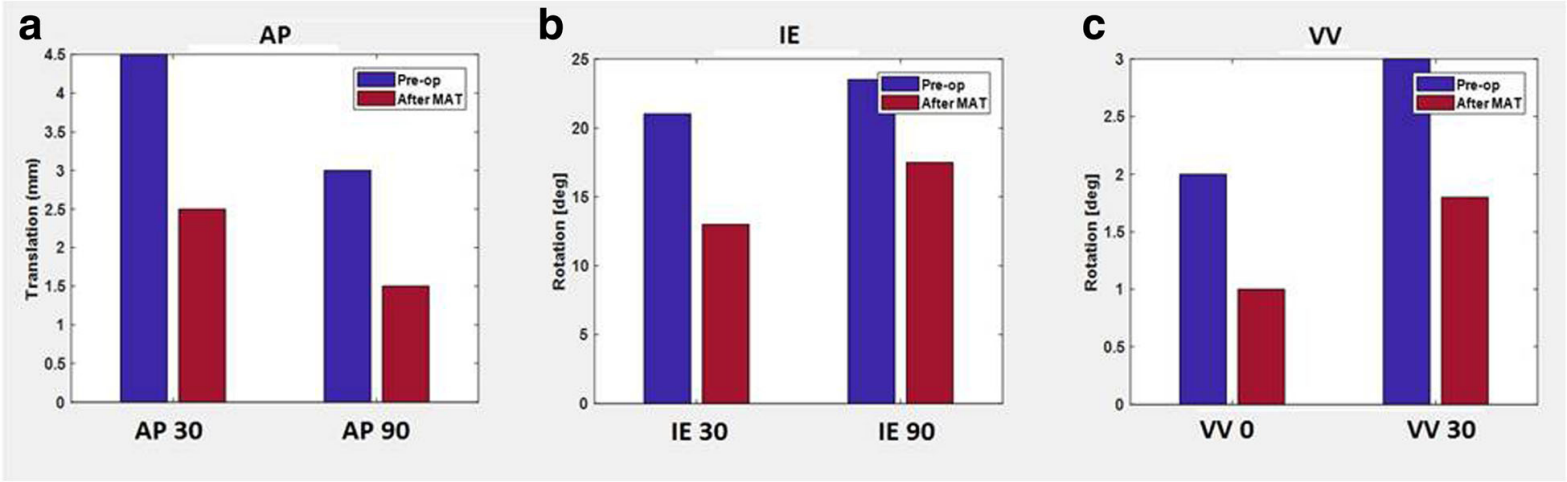
Laxity evaluation: AP30 and AP90 (**a**), IE30 and IE90 (**b**), VV0 and VV30 (**c**) for Lateral MAT

AP displacement at 30° of flexion was 4.5 mm at the basal state and 2.5 mm after MAT while the AP displacement at 90° of flexion was 3 mm at the basal state and 1.5 mm after MAT. IE rotation at 30° of flexion was 21° at the basal state and 13° after MAT while IE rotation at 90° of flexion was 23.5° at the basal state and 17.5° after MAT. VV rotation at 0° of flexion was 2.0° at the basal state and 1° after MAT, while VV rotation at 30° of flexion was 3° at the basal state and 1.8° after MAT (Fig. 8).

## Discussion

The aim of the present work was to evaluate the biomechanical role of MAT on knee laxity, through both a narrative review of the literature and two case studies, intraoperatively assessed with surgical navigation system.

Due to the biomechanical effect of both load absorbing and secondary stabilizer, meniscal repair should be the treatment of choice in case of lesions. However, since most of lesions are irreparable and require partial or even subtotal meniscectomies, MAT could be considered a viable and effective treatment for post-meniscectomy syndrome. In fact, MAT demonstrated to be biomechanically effective in improving knee stress distribution and reducing laxities (Nyland et al., 2018). For these reasons, the International Meniscus Reconstruction Experts Forum (IMREF) suggested MAT also in the setting of revision ACL reconstruction when meniscal deficiency is considered responsible of primary reconstruction failure, (Getgood et al., 2017) due to its effect of secondary stabilizer. Case series of primary or revision ACL reconstruction combined with MAT demonstrated satisfactory clinical outcomes and good knee stability (Zaffagnini et al., 2018).

The preliminary results of the in-vivo evaluation of MAT with computer navigation offered some interesting considerations. The MAT was in fact able to decrease knee laxity both in a medial and lateral meniscus-deficient knee, thus suggesting a synergic role with ACL in laxity, especially in resisting anterior tibial translation.

This study has some limitations. The two cases of MAT here described were performed in different settings. The medial MAT, since performed in an ACL and medial meniscus deficient knee, just before ACL reconstruction, is not able to provide information on the contribution of meniscal replacement to an intact-ACL knee. Differently, the lateral MAT was performed in an intact-ACL knee. Thus, it was not possible to compare the results from the two assessments, but we could only evaluate the pure role of the meniscus on the knee laxity. However, the different role on knee laxity of the medial and lateral meniscus would have created a bias in the comparison.

Furthermore, the kinematic evaluation has been performed manually rather than with mechanical devices and standardized forces. However, the senior surgeon has more than 10-year experience with surgical navigation of ACL reconstruction and his high reliability in manual assessment has already been demonstrated (Lopomo et al., 2009; Martelli et al., 2007). Another limitation is represented by the absence of contralateral knee laxity evaluation. This evaluation would have been useful to assess the real side-to-side difference in laxity and thus its relative reduction after MAT. Anyway, even if this practice is commonly performed in cadaveric studies, it would have been unethical in-vivo.

Moreover, these data represent a unique and preliminary experience of in-vivo assessment of MAT, thus they should be confirmed in larger series.

## Conclusions

Meniscal Allograft Transplantation represents a valuable solution to improve the overall biomechanics of the knee joint and help to restore a good clinical condition, when associated with ACL replacement. The in-vivo kinematic evaluation here described confirmed the importance of MAT in reducing knee laxity for the two presented cases, particularly regarding the AP translation for the medial MAT and IE rotation for the lateral MAT. Further in-vivo studies may help to better assess the role of MAT in combination with ACL replacement and give insights for a better comprehension of the contribute of meniscal replacement in knee surgery.

## Data Availability

The datasets used and analyzed during the current study are available from the corresponding author on reasonable request.

## Abbreviations

ACL: Anterior Cruciate Ligament
AP: anterior-posterior
BMI: Body Mass Index
IE: Internal-External
IMREF: International Meniscus Reconstruction Experts Forum
MAT: Meniscus allograft transplantation
MRI: Magnetic Resonance Imaging
OA: Osteoarthritis
PCL: Posterior Cruciate Ligament
VV: Varus-Valgus
